# Marfan Syndrome Associated With Acute Myocardial Infarction in the First Trimester of Pregnancy

**DOI:** 10.7759/cureus.59182

**Published:** 2024-04-28

**Authors:** Ioannis Korkontzelos, Anna Kotsia, George Mpourazanis, Angelos Natsios, Pantelina-Danai Korkontzelou, Pavlos Karanikis, Evgenia Pappa, Petros Papalexis

**Affiliations:** 1 Department of Obstetrics and Gynecology, Ioannina State General Hospital “G. Chatzikosta”, Ioannina, GRC; 2 Department of Cardiology, Ioannina State General Hospital “G. Chatzikosta”, Ioannina, GRC; 3 Department of Medicine, Medical University of Sofia, Sofia, BGR; 4 Department of Endocrinology, First Department of Internal Medicine, Laiko General Hospital, National and Kapodistrian University of Athens, Athens, GRC; 5 Department of Medicine, Faculty of Health Sciences, Aristotle University of Thessaloniki, Thessaloniki, GRC

**Keywords:** cardiopathy in pregnancy, marfan disease, pregnancy, acute myocardial infarction, marfan's syndrome

## Abstract

Marfan syndrome (MFS) is a rare hereditary connective tissue disorder with autosomal dominant inheritance associated with an increased risk of cardiovascular complications due to connective tissue fragility. Acute myocardial infarction during pregnancy is also a rare event associated with poor maternal and fetal outcomes. Herein, we report a case of a 30-year-old pregnant woman with a known history of MFS. The patient had been treated surgically for an ascending aorta aneurysm and mechanical prosthetic aortic valve repair. She presented at 12 weeks of gestation with severe chest pain, which proved to be acute myocardial infarction. This is believed to be the first case of this complex medical condition presented in the first trimester of pregnancy.

## Introduction

Marfan syndrome (MFS) is a hereditary connective tissue disorder with autosomal dominant inheritance, which is caused by a mutation in the gene encoding fibrillin-1 (FBN1). This gene is sited on chromosome 15q21, and the disease affects approximately 1/5000 people. Heterozygous mutations in the transforming growth factor beta 2 receptor (TGFBR2) gene sited on chromosome 3p24.2-25 have also been identified in other Marfan-like syndromes [1]. Furthermore, 15%-25% of the syndrome is due to spontaneous mutations [2].

In MFS, cardiovascular complications present in 80% of the patients, including aortic dilatation, aortic incompetence, and mitral or tricuspid valve prolapse with or without regurgitation, followed by abnormalities of the skeletal, ocular, and dural system [3]. Major causes of death include aortic aneurysm dissection and rupture. During pregnancy, the reported incidence of severe cardiovascular events is 3%-7%, and the overall risk of fatal complications is approximately 1% [4].

Acute myocardial infarction (AMI) during pregnancy is also a major cause of morbidity and mortality associated with poor maternal and fetal outcomes. It is considered a rare event with an increased risk of three- to four-fold and an estimated incidence of 1/10000 deliveries. In general, risk factors for AMI include women above the age of 30, cigarette smoking, diabetes, dyslipidemia, multiparity, the third trimester of pregnancy, hypertension, preeclampsia or eclampsia, transfusion, thrombophilia, and postpartum infections [5].

## Case presentation

Α 30-year-old pregnant woman, primigravida, nonsmoker, with a known history of MFS presented to the emergency unit at 12 weeks of gestation with acute severe chest pain. From the medical history, we noted a surgically treated ascending aorta aneurysm accompanied by mechanical prosthetic aortic valve repair. Moreover, three months before the incidence, she was diagnosed with thrombophilia, which included six gene mutations (factor XII, plasminogen activator inhibitor-1 [PAI-1], glycoproteins IIIA [GPIIIA], methylenetetrahydrofolate reductase [MTHFR] with homozygosity, angiotensin-converting enzyme [ACE], and apolipoprotein-E [APO-E]. The patient had been treated with acenocoumarol as antithrombotic therapy, which was discontinued during pregnancy by the attending cardiologist and replaced with enoxaparin 6000 IU b.i.d. at the time of presentation. We also noted from the medical history that the patient did not attend counseling before conception and that she suffered COVID-19 infection one month ago without serious complications despite being fully vaccinated.

**Figure 1:**
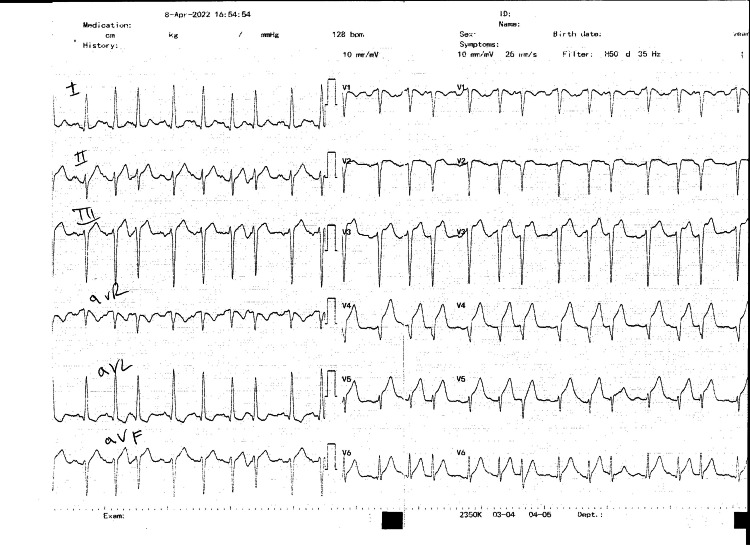
Twelve-lead electrocardiogram showing ST-segment elevation in leads V2-V6 and depression in leads I and aVL aVL: augmented Vector Left.

On admission, the patient presented with chest pain, was tachycardic but hemodynamically stable, had a heart rate of 100/bpm, blood pressure of 108/71 mmHg, respiratory rate of 20/min, SpO_2 _of 99%, and a temperature of 36.4°C. An electrocardiogram revealed ST elevation in leads V2-V6, and the diagnosis of anterior myocardial infarction-STEMI was established (Figure 1). She was administered acetylsalicylic acid (ASA) 500 mg and methylprednisolone 125 mg bolus.

**Figure 2:**
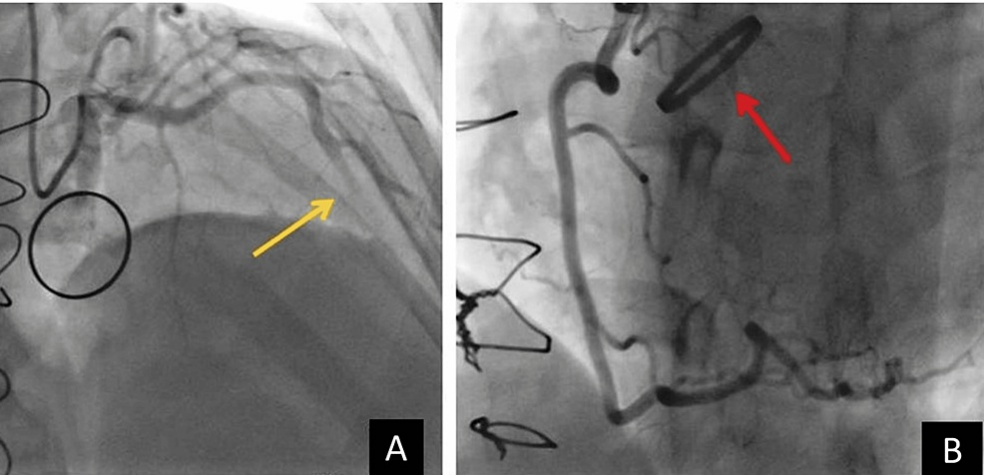
(A) Angiography of the left anterior descending artery (LAD) with total occlusion of the mid-part (yellow arrow). (B) Angiography of the normal right coronary artery (RCA) and normal mechanic aortic valve function (red arrow).

After admission, emergent coronary angiography revealed total occlusion in the mid-left anterior ascending artery (LAD), while the left circumflex artery (LCx) and right coronary (RCA) artery had normal opacification (Figure 2). The valve had a normal function in radioscopy (Figure 2, Panel B).

**Figure 3:**
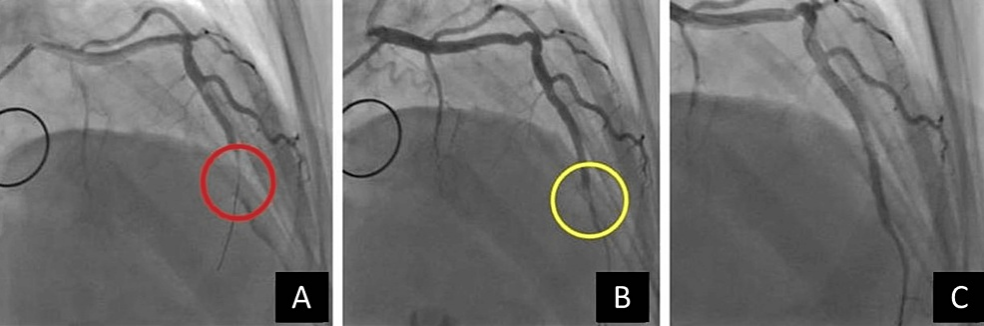
(A) Left anterior descending artery (LAD) after first wiring (red circle). (B) Left anterior descending artery (LAD) after the first stent implantation (yellow circle). (C) Left anterior descending artery (LAD) after the second stent implantation.

Primary percutaneous coronary angioplasty with two drug-eluting overlapping stents was performed in the occluded artery (Figure 3). Laboratory results during hospitalization are listed in Table 1.

**Table 1:** Laboratory diagnostic follow-up of the patient during hospitalization

Parameters	Day 0 admission	Day 1	Day 3	Day 5	Day 7	Reference range
Hematocrit	43.8	40.2	40.5	38.5	38.5	36%-52%
Hemoglobin	15.2	13.6	13.7	13.3	13.3	11.8-17.8 g/dl
White blood cells	17.900	24.890	16900	14100	12800	4-11 k/mL
Platelet	468	429	390	386	420	140-450 k/mL
C-reactive protein	0.63	0.60	2.30	5.93	7.97	0-0.80 mg/dL
Troponin	2728	68973	27411	12878	3014	0-15.6 ng/L
Cholesterol	191	192	188	176	177	120-220 mg/dl
High-density lipoprotein	43	44	40	36	35	45-65 mg/dl
Triglycerides	88	88	87	84	84	30-160 mg/dl
Lactate dehydrogenase	459	1550	1303	825	680	120-230 IU/L
Creatine kinase	478	2620	264	112	77	0-220 IU/L
Creatine kinase-MB	76	403	92	20	19	0-23 U/L
Thyroid-stimulating hormone	2.58	2.56	2.59	-	-	0.38-5.33 μIU/ml
Free thyroxine 4	1.02	1.03	1.06	-	-	0.6-1.3 μIU/ml
Serum aspartate transaminase/alanine transaminase	58/30	335/91	70/62	39/55	24/33	5-33 U/L
International normalized ratio	0.97	1.05	1.06	1.06	1.07	1-1.3
D-dimers	0.68	1.23	-	-	-	-0.5 mg/L

After the intervention, the echocardiography showed moderately reduced left ventricular systolic function (ejection fraction ~ 40%) with akinesis of the apex, apical inferior, and mid to apical septal wall segments as well as hypokinesis of the anterior wall. During hospitalization, the patient was treated with ASA 100 mg o.d., clopidogrel 75 mg o.d., enoxaparine 8000 IU b.i.d., bisoprolol 2.5 mg o.d., magnesium tab X 2, and folic acid 5 mg X 1. She remained stable and asymptomatic for the rest of her hospitalization and was discharged after seven days in good condition. At 13+4 weeks of gestation, she underwent the first-trimester ultrasound scan for chromosomal abnormalities with simultaneous chorionic villus sampling. The results revealed severe fetal expression of the syndrome, and the patient decided to terminate the pregnancy.

## Discussion

MFS is a rare disorder affecting elastin, a connective tissue responsible for vascular and organ elasticity [2]. The current diagnosis is based on clinical assessment and the revised 2010 Ghent nosology, which emphasizes cardiovascular abnormalities more. During pregnancy, there is an increased risk of maternal and fetal complications caused by cardiovascular adaptations in response to increased blood volume-oxygen demands and decreased tissue elasticity of the syndrome [1,6].

AMI related to pregnancy is also a rare event, associated with atherosclerosis, coronary artery dissection or spasm, aortic valve stenosis, aortic prosthetic valve thrombosis, sickle cell disease, cocaine usage, and pheochromocytoma. The maternal mortality rate is reported to be 11% occurring in most of the patients at the time of infarction or within two weeks [5].

The risk of coronary thrombosis during pregnancy without underlying atherosclerosis is reported as high as 17%. It could be explained by the changes in the coagulation and fibrinolytic system causing a hypercoagulopathy state [5]. In general, the most common cause of AMI in obstetrics is spontaneous coronary artery dissection (SCAD), especially in the peripartum period, reaching 50% of the cases, and the mortality is twice as high [5]. SCAD is defined as a spontaneous tear in a coronary artery that is not associated with atherosclerosis, trauma, or α medical intervention. In particular, apart from pregnancy, risk factors for SCAD include female sex, severe emotional or physical stress, oral contraceptives, autoimmune vasculitis, underlying blood vessel diseases such as fibromuscular dysplasia, and connective tissue diseases such as MFS or Ehlers-Danlos syndrome [7].

Counseling and management of MFS patients should start before conception. There is a high risk of transmission of the syndrome (>50%), and severe expression could occur even in relatively mild cases. Almost 80% of these patients could present with cardiovascular complications. The expected rate of aortic dissection may range from 3% in women with aortic diameter <40 mm to as much as 10% in patients with aortic diameter >40 mm [8]. Thromboembolic events were also reported in women with mechanical prosthetic heart valve repair treated with unfractionated heparin [9].

The obstetrical complication rate is 40%, with a five-fold increased risk of venous thromboembolism. Postpartum hemorrhage (PPH) is double that of the general obstetric population. In the peripartum period, disseminated intravascular coagulation (DIC), pneumothorax, arrhythmias, cardiomyopathy, heart failure, and cardiac arrest could also occur. Neonatal complications include premature rupture of membranes in 15% and neonatal mortality in 7% [6,8].

MFS patients should undergo careful cardiovascular evaluation, including both transthoracic echocardiogram (TTE) and transesophageal echocardiogram (TEE), for the assessment of proximal and distal aortic diameter and the valvular-cardiac function. Evaluation of the distal aorta is especially important in patients with dilatation or a history of surgical repair of the proximal aorta [8]. Women with an aortic diameter > 40 mm are strongly discouraged from becoming pregnant without prior surgery. However, aortic valve repair does not safeguard against future dissection during pregnancy or in the postpartum period [2,10].

Computed tomography (CT) and magnetic resonance (MR) angiography (CTA and MRA, respectively) remain the mainstay for postoperative imaging modalities for surveillance of the aorta in MFS and can demonstrate possible complications [11]. CTA and MRA could be also used for a precise assessment of the aortic size and anatomy before conception [10].

In pregnancy, MFS women should undergo regular imaging with echocardiography. Echocardiography is recommended every trimester for normal aortic roots (<4 mm) and every eight weeks for dilated roots (>4 mm). After birth, they should undergo a repeat echo before discharge and six months after postpartum. Treatment should include tobacco cessation and β-blockers to control hypertension and reduce the risk of dissection [10]. Metoprolol and labetalol, which possess the activity of both β- and α-adrenergic receptor blockers, are safe and efficient during pregnancy and should be administered to alleviate aortic wall stress and decrease the risk of aorta enlargement. Atenolol has been associated with fetal growth restriction. If β-blockers are contraindicated, calcium channel blockers may be administrated especially nifedipine [2,5,10].

Our case was presented in the first trimester of pregnancy; however, most of the AMI cases occur in the third trimester or the postpartum period. Diagnostic criteria are based on symptoms, electrocardiographic changes, and cardiac markers. Symptoms include chest pain, dyspnea, tachypnea, fatigue, dizziness, palpitations, and syncope. Electrocardiograms could present with Q waves or ST-segment changes. Troponin-I levels remain the most important examination. Unlike creatine kinase-MB (CKMB) which elevates with uterine contractions, troponin remains the standard for evaluating cardiac ischemia as levels are not affected by pregnancy. However, elevated troponin-I could be also seen in pericarditis, myocarditis, demand ischemia, and acute pulmonary embolism [5]. In the third trimester, ST-elevation myocardial infarction (STEMI) presents in 25%, and non-ST elevation myocardial infarction (NSTEMI) presents in 32%, while in the postpartum period, STEMI and NSTEMI are present in 45% and 55%, respectively. The anterior wall is most commonly involved in 69% of the patients, the inferior wall is involved in 27%, and the lateral wall is involved in 4% [12].

Marfan patients who suffer myocardial infarction during pregnancy should be treated with percutaneous coronary intervention (PCI) and thrombolytic therapy. PCI is considered superior to thrombolytic therapy alone, provided it can be performed within 12 hours after symptom onset. Low molecular weight heparin and antiplatelet agents like low-dose aspirin (60-150 mg) should be administered. High-dose aspirin should be avoided due to the increased risk of maternal and fetal hemorrhage. Clopidogrel should be used only when strictly needed especially after stent implantation. HMG-CoA reductase inhibitors are used for the reduction of low-density lipoproteins; however, available research is inadequate for its safety in pregnancy [5,8].

The role of hereditary thrombophilia and myocardial infarction in pregnancy is still under consideration since the risk of arterial thrombosis is less well-defined. Thrombophilia is a risk factor for venous thromboembolism, but an association between antiphospholipid syndrome and AMI has been also reported. Furthermore, case reports noting the association between factors V Leiden, V R2-mutation, MTHFR C677T, and AMI have been also stated. However, large studies showed no significant role of thrombophilia in arterial disease indicating that genetic or environmental agents are more important in the pathogenesis of this entity [13,14].

Previous investigations have reported that viral infections could lead to microcirculatory dysfunction, microvascular injury, and the creation of microthrombi. Patients with coronavirus have increased procoagulants, which correlate with acute-phase reactants. This inflammatory response could have a major contribution to thrombogenesis [15]. In this regard, recent data have demonstrated the possible association between COVID-19 and AMI. In a single-center observational study, patients with STEMI managed with primary PCI showed a higher thrombus burden and a higher rate of multivessel or stent thrombosis in COVID-19 patients compared with non-COVID patients [16]. Furthermore, long COVID or post-acute sequelae of coronavirus, defined as the presence of symptoms for more than three months or the manifestation of cardiovascular events including myocardial inflammation, myocardial infarction, right ventricular dysfunction, and arrhythmias, have been also reported [17]. Pregnant women with COVID-19 infection and prior cardiovascular disease are deemed at high risk for AMI. Evidence of myocarditis, persistent myocardial infarction, inflammation, or subclinical myocardial injury could be seen months after acute COVID-19 infection [18].

## Conclusions

This is believed to be a rare report of MFS associated with AMI during the first trimester of pregnancy. In the available literature, only one other case of a pregnant woman with MFS who suffered from myocardial infarction in the postpartum period due to spontaneous dissection of the left anterior descending coronary artery has been reported. Whether the cause of this event was single or multifactorial (MFS, pregnancy, thrombophilia, and coronavirus) will remain unanswered. Overall, this case highlights the importance of preconception genetic and cardiac counseling for patients with MFS. While clinicians should focus on aortic dissection or rupture, rare events like myocardial infarction should also be taken into consideration. Women with major risk factors for cardiopathy in pregnancy require close and constant surveillance and individual treatment from a multidisciplinary medical team.
